# Prospective Evaluation of Different Methods for Volumetric Analysis on [^18^F]FDG PET/CT in Pediatric Hodgkin Lymphoma

**DOI:** 10.3390/jcm11206223

**Published:** 2022-10-21

**Authors:** Egesta Lopci, Caterina Elia, Barbara Catalfamo, Roberta Burnelli, Valli De Re, Lara Mussolin, Arnoldo Piccardo, Angelina Cistaro, Eugenio Borsatti, Pietro Zucchetta, Maurizio Bianchi, Salvatore Buffardi, Piero Farruggia, Alberto Garaventa, Alessandra Sala, Luciana Vinti, Christine Mauz-Koerholz, Maurizio Mascarin

**Affiliations:** 1 Nuclear Medicine Unit, IRCCS—Humanitas Research Hospital, Via Manzoni 56, 20089 Rozzano, Italy; 2 AYA and Pediatric Radiotherapy Unit, Centro di Riferimento Oncologico di Aviano (CRO), IRCCS, 33081 Aviano, Italy; 3 Nuclear Medicine Unit, University Hospital “Mater Domini, 88100 Catanzaro, Italy; 4 Pediatric Onco-Hematologic Unit, University Hospital S. Anna, 44121 Ferrara, Italy; 5 Immunopathology and Cancer Biomarkers Unit, Department of Translational Research, Centro di Riferimento Oncologico di Aviano (CRO), IRCCS, 33081 Aviano, Italy; 6 Pediatric Hemato-Oncology Clinic, Department of Women’s and Children’s Health, University of Padua, 35128 Padua, Italy; 7 Institute of Pediatric Research-Fondazione Città della Speranza, 35127 Padua, Italy; 8 Department of Nuclear Medicine, Galliera Hospital, 16128 Genoa, Italy; 9 Nuclear Medicine Division, Salus Alliance Medical, 16128 Genoa, Italy; 10 Nuclear Medicine Department, Centro di Riferimento Oncologico di Aviano (CRO), IRCCS, 33081 Aviano, Italy; 11 Nuclear Medicine Department, Padova University Hospital, 35128 Padua, Italy; 12 Onco-Hematology Division, Regina Margherita Hospital, 10126 Torino, Italy; 13 Department of Oncology, Hospital Santobono-Pausilipon, 80123 Naples, Italy; 14 Department of Pediatric Onco-Hematology, A.R.N.A.S. Ospedali Civico, 90127 Palermo, Italy; 15 Pediatric Oncology Unit, I RCCS G.Gaslini Hospital, 16147 Genoa, Italy; 16 Pediatric Division, Hospital San Gerardo, 20900 Monza, Italy; 17 Department of Pediatric Hematology and Oncology, Ospedale Bambino Gesù, IRCSS, 00165 Rome, Italy; 18 Pädiatrische Hämatologie und Onkologie, Zentrum für Kinderheilkunde der Justus-Liebig-Universität Gießen, 35392 Giessen, Germany; 19 Medizinische Fakultät der Martin-Luther-Universität Halle-Wittenberg, 06120 Halle, Germany

**Keywords:** FDG PET, Hodgkin’s lymphoma, pediatric, volumetric analysis, response assessment, interim evaluation, method comparison

## Abstract

Rationale: Therapy response evaluation by ^18^F-fluorodeoxyglucose PET/CT (FDG PET) has become a powerful tool for the discrimination of responders from non-responders in pediatric Hodgkin lymphoma (HL). Recently, volumetric analyses have been regarded as a valuable tool for disease prognostication and biological characterization in cancer. Given the multitude of methods available for volumetric analysis in HL, the AIEOP Hodgkin Lymphoma Study Group has designed a prospective analysis of the Italian cohort enrolled in the EuroNet-PHL-C2 trial. Methods: Primarily, the study aimed to compare the different segmentation techniques used for volumetric assessment in HL patients at baseline (PET1) and during therapy: early (PET2) and late assessment (PET3). Overall, 50 patients and 150 scans were investigated for the current analysis. A dedicated software was used to semi-automatically delineate contours of the lesions by using different threshold methods. More specifically, four methods were applied: (1) fixed 41% threshold of the maximum standardized uptake value (SUVmax) within the respective lymphoma site (V41%), (2) fixed absolute SUV threshold of 2.5 (V2.5); (3) SUVmax(lesion)/SUVmean liver >1.5 (Vliver); (4) adaptive method (AM). All parameters obtained from the different methods were analyzed with respect to response. Results: Among the different methods investigated, the strongest correlation was observed between AM and Vliver (rho > 0.9; *p* < 0.001 for SUVmean, MTV and TLG at all scan timing), along with V2.5 and AM or Vliver (rho 0.98, *p* < 0.001 for TLG at baseline; rho > 0.9; *p* < 0.001 for SUVmean, MTV and TLG at PET2 and PET3, respectively). To determine the best segmentation method, we applied logistic regression and correlated different results with Deauville scores at late evaluation. Logistic regression demonstrated that MTV (metabolic tumor volume) and TLG (total lesion glycolysis) computation according to V2.5 and Vliver significantly correlated to response to treatment (*p* = 0.01 and 0.04 for MTV and 0.03 and 0.04 for TLG, respectively). SUVmean also resulted in significant correlation as absolute value or variation. Conclusions: The best correlation for volumetric analysis was documented for AM and Vliver, followed by V2.5. The volumetric analyses obtained from V2.5 and Vliver significantly correlated to response to therapy, proving to be preferred thresholds in our pediatric HL cohort.

## 1. Introduction

Hodgkin lymphoma (HL) is one of the most frequent yet curable hematological malignancies in children [1]. Therefore, it appears to be of paramount importance to optimize therapeutic effect and minimize subsequent long-term risks while maintaining high cure rates. For this reason, it appears necessary to customize therapy for each patient based on the pre-treatment prognostic factors and intermediate assessments of disease response. Over the past few decades, ^18^F-fluorodeoxyglucose PET/CT (FDG PET) has played an increasingly central role for staging, management and follow-up of various pediatric malignancies [2,3,4,5,6,7]. In pediatric HL, therapy response evaluation by means of [^18^F]FDG PET has become a powerful tool for the discrimination of adequate responders from inadequate responders [8,9,10]. According to international standards, the Deauville five-point scale is considered the visual method of choice for discriminating responses in patients with lymphoma [11,12,13,14]. In order to extend the Deauville score to a continuous scale and limit visual misinterpretation, the German pediatric HL group proposed the use of qPET in 2014 [15,16,17]. This quantitative method was applied in the current EuroNet-PHL-C2 clinical study, in which therapy was adapted based on the FDG PET result after two cycles of chemotherapy [18,19].

Nevertheless, most of the studies were conducted in adolescent patients, and although PET appears to have similar diagnostic performance in the pediatric population, studies in the pediatric cohort are limited. Therefore, the criteria for the definition of adequate or inadequate response in children with HL are still being discussed [16].

Recently, volumetric analyses have been regarded as a valuable tool for disease prognostication and biological characterization in cancer. Effectively, in addition to semiquantitative parameters (i.e., SUVmax, SUVmean, SUVpeak), [^18^F]FDG PET provides three-dimensional disease volume measurements and metabolic activity, such as metabolic tumor volume (MTV) and total lesion glycolysis (TLG). These functional measurements of tumor volume provide additional prognostic information beyond the classical risk factors that include a unidimensional measurement of tumor bulk [20]. A high total-body tumor burden, defined by these parameters, is associated with poorer prognosis in adults with various lymphomas, including HL [20,21,22,23,24,25,26,27,28,29,30,31,32,33].

Given the multitude of methods available for volumetric analysis in HL, the AIEOP Hodgkin Lymphoma Study Group has designed a prospective analysis of the Italian cohort enrolled in the EuroNet-PHL-C2 trial. The aim of this study is to investigate different segmentation techniques used for volumetric assessment in HL patients at baseline and during the course of therapy and to compare the parameters obtained by the different methods, relating them to the response.

## 2. Materials and Methods

### 2.1. Study Population

The population analyzed in the current study was obtained from the Italian cohort of patients treated according to the EuroNet-PHL-C2 trial [34] and enrolled in the prospective parallel study promoted by the AIEOP Hodgkin Lymphoma Study Group following Amendment Nr. 04, dated July 31, 2017 [34]. The study was approved by AIFA (Agenzia Italiana del Farmaco) on March 9, 2018. Written informed consent was obtained from all subjects or their legal representatives before inclusion. In accordance with the EuroNet-PHL-C2 trial, the study population comprised pediatric patients with histologically confirmed classical HL in intermediate and advanced treatment level, evaluated with FDG PET at baseline (PET1), after two cycles of induction therapy (PET2) and after the end of chemotherapy (PET3), in case of PET2 positivity [34]. Primarily, the present study aimed to compare the different segmentation techniques used for volumetric assessment in HL patients at baseline and during the course of therapy. Therefore, an overall population of 50 patients (150 scans) were investigated across 17 Italian AIEOP Centers.

Principal characteristics of the study population are shown in Table 1.

### 2.2. Volumetric Assessment

**Figure 1:**
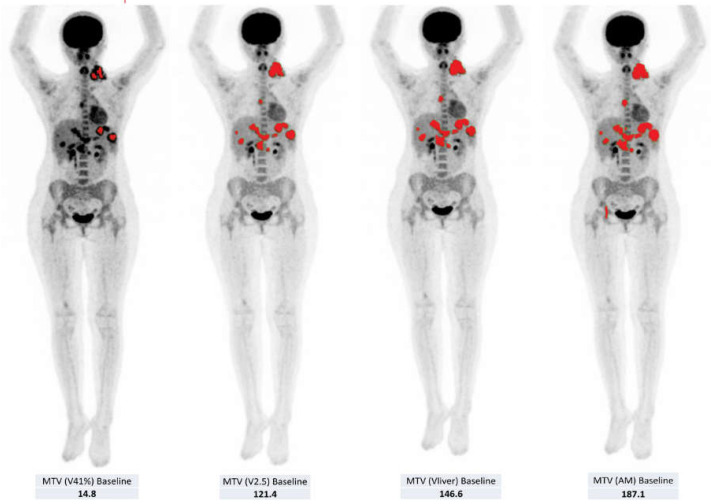
MIP (maximal intensity projection) image illustration of the four segmentation techniques applied in our study. From left to right: fixed 41% threshold (V41%), fixed absolute SUV threshold of 2.5 (V2.5), SUVmax(lesion)/SUVmean liver >1.5 (Vliver) and adaptive method (AM). Corresponding MTV (metabolic tumor volumes) at baseline are shown beneath each MIP image. The areas highlighted in red in the figures indicate the contours of the malignant lesions delineated by each segmentation technique.

Imaging protocol for FDG PET scans was compliant with the requirements of the EuroNet-PHL-C2 trial, which were in accordance with the EANM guidelines for patient preparation, data acquisition and image reconstruction [35] to avoid discrepancies between the different PET tomographs used in the various AIEOP Centers involved in the study. For the semi-quantitative and volumetric analyses of the FDG PET scans, the Local Image Features Extraction (LIFEx) freeware (http://www.lifexsoft.org) was used to semi-automatically delineate contours of the lesions by using different threshold methods (Figure 1). More specifically, we utilized four methods for volumetric assessment of pediatric HL [36]: (1) fixed 41% threshold of SUVmax within the respective lymphoma site (V41%), (2) fixed absolute SUV threshold of 2.5 (V2.5), (3) SUVmax(lesion)/SUVmean liver > 1.5 (Vliver) and (4) adaptive method (AM) [35]. This was computed as [0.15 × I(mean)] + I(background), where I(mean) is calculated as the mean intensity of all pixels surrounded by the 70% Imax isocontour within the tumor while I(background) is defined as SUVmean of the liver. [36]. The semi-quantitative parameters retrieved from the different analyses comprised metabolic tumor volume (MTV) and total lesion glycolysis (TLG = MTV × SUVmean) at baseline and during the course of therapy, as well as SUVmax, determined as the pixel with the highest uptake value; SUVmean, as the mean value of uptake; and SUVpeak, corresponding to the average value of uptake in a VOI (volume of interest) of 1ml that surrounds the pixel with the highest activity.

### 2.3. Response Classification

According to the EuroNet-PHL-C2 trial, patients are classified into Adequate Response (AR) and Inadequate Response (IR). In the current study, we subdivided treatment response based on the Deauville-5-points-scale (DS) into the following: DS 1 = no uptake; DS 2 = uptake ≤ mediastinum; DS 3 = uptake > mediastinum but ≤ liver; DS 4 = uptake moderately more than liver uptake, at any site; DS 5 = markedly increased uptake at any site and new sites of disease [37]. The percentage variation of all semiquantitative parameters (i.e., SUVmax, SUVmean, SUVpeak, MTV and TLG) from baseline (PET1) to early (PET2) and late assessment (PET3) was computed. Since the aim of the present study was not to provide treatment outcomes from the EuroNet-PHL-C2 trial but rather select the best threshold method for volumetric assessment, we did not display the responses obtained from the Italian cohort and used the dichotomization according to DS only to validate the robustness of each method under investigation.

### 2.4. Statistical Analysis

Descriptive statistics comprised conventional metrics (mean, median, range). The different threshold methods used to outline lymphoma lesions were compared by the Pearson correlation coefficient, linear regression, Bland–Altman and logistic regression. Linear regression was applied to determine the relationship between response to treatment at early (PET2) and late evaluation (PET3), defined according to Deauville score (classified into DS 2, DS 3, DS 4 and DS 5) and all other variables classified with the different volumetric thresholds (i.e., V41%, V2.5, Vliver and AM). Statistical significance was set for a *p* value < 0.05.

## 3. Results

### 3.1. Semi-Quantitative and Volumetric Analyses

The semi-quantitative and volumetric analyses obtained from the different threshold methods at baseline showed the following results for SUVmean, with regards to median values: V2.5 (5.2), V41% (8.5), Vliver (2) and AM (5 4.75), respectively. The corresponding MTV values resulted as follows: V2.5 (334.2), V41% (82.9), Vliver (423.2) and AM (433.5), respectively. The TLG median values were V2.5 (1639.8), V41% (570), Vliver (1745.75) and AM (1782.2), respectively. The values obtained at PET2 assessment for SUVmean resulted in V2.5 (3.1), V41% (2.7), Vliver (2) and AM (2.9), respectively. The corresponding PET2 MTV values resulted in V2.5 (2.15), V41% (5.95), Vliver (1.9) and AM (6.5), respectively. The PET2 TLG median values were V2.5 (9.45), V41% (19.85), Vliver (7.9) and AM (20.6), respectively. Lastly, the median values obtained at PET3 for SUVmean, MTV and TLG were all cleared to zero.

### 3.2. Comparison of the Parameters according to the Different Methods

**Figure 2:**
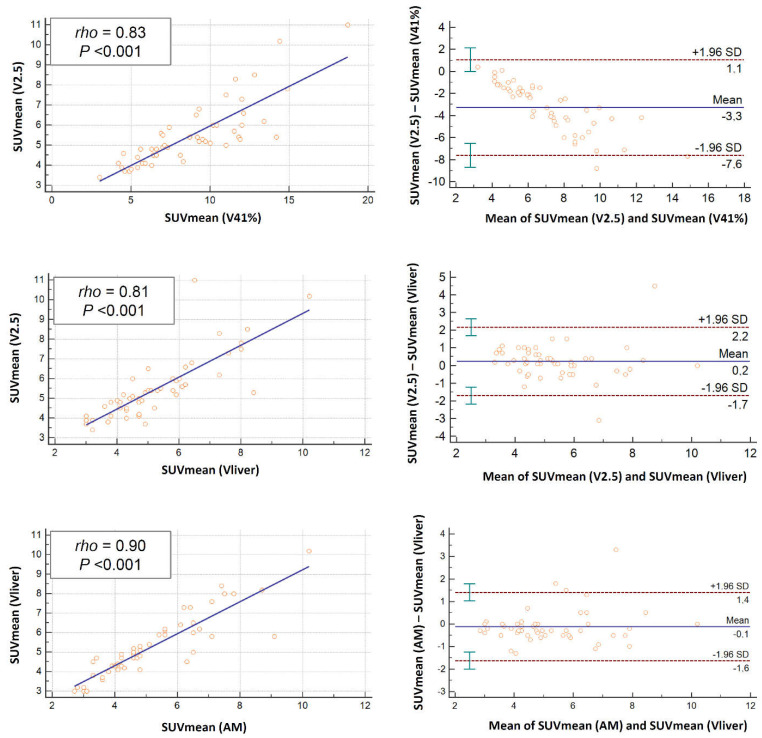
Scatter plots for linear regression with corresponding correlation coefficient (rho) and p-values related to the different segmentation techniques used (**left columns**); Bland-Altman plots (**right columns**) for baseline SUVmean values showing mean and standard deviations (SD) lines, revealing some outliners in all methods. The closer the data with respect to the mean lines and the lower the variation between SD, the high the comparability of the methods. Herein, the calculated limits of agreement are substantial especially for the V41% method.

**Figure 3:**
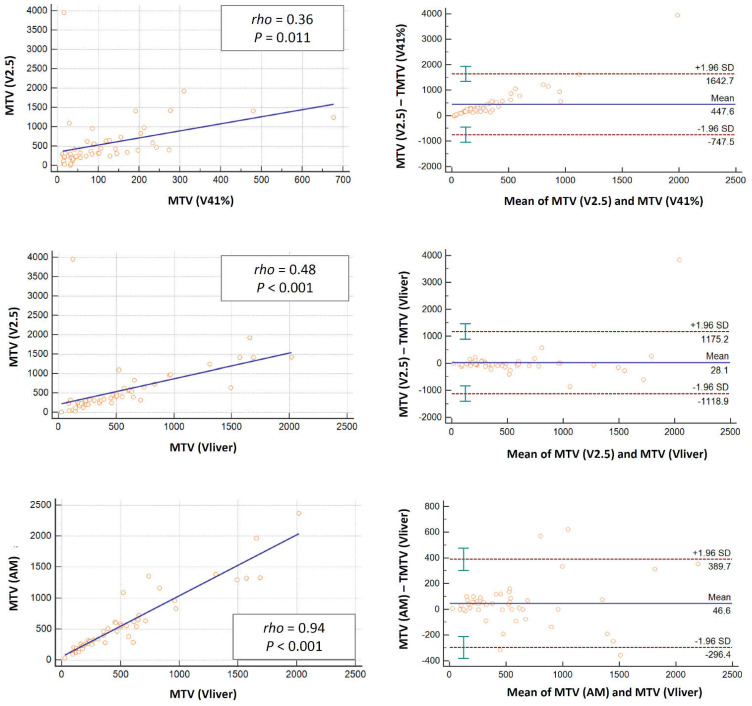
Scatter plots for linear regression with corresponding correlation coefficient (rho) and p-values related to the different segmentation techniques used (**left columns**); Bland-Altman plots (**right columns**) for baseline MTV values showing mean and standard deviations (SD) lines, revealing some outliners in all methods. The closer the data with respect to the mean lines and the lower the variation between SD, the high the comparability of the methods. Herein, the calculated limits of agreement are substantial especially for the V41% method.

**Figure 4:**
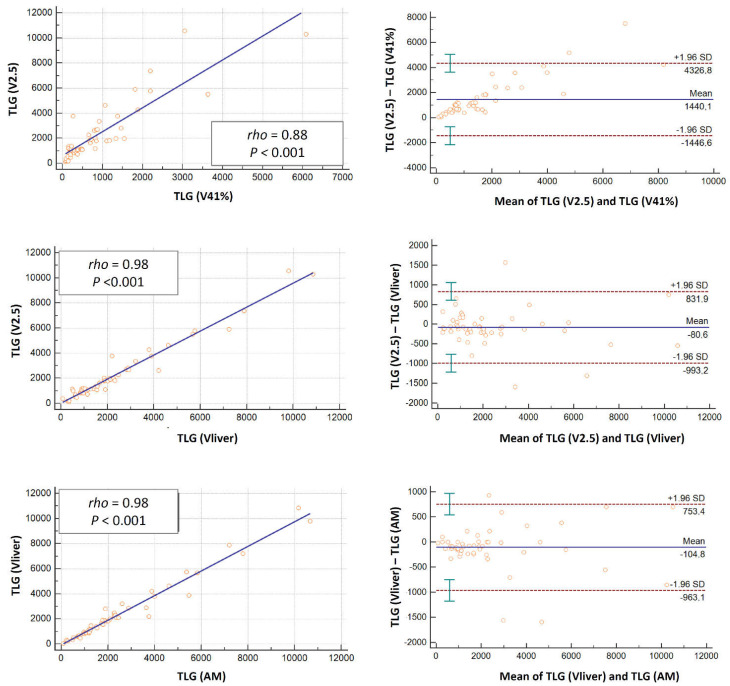
Scatter plots for linear regression with corresponding correlation coefficient (rho) and p-values related to the different segmentation techniques used (**left columns**);Bland-Altman plots (**right columns**) for baseline TLG values showing mean and standard deviations (SD) lines, revealing some outliners in all methods. The closer the data with respect to the mean lines and the lower the variation between SD, the high the comparability of the methods. Herein, the calculated limits of agreement are substantial especially for the V41% method.

The linear regression allowed us to compare the semi-quantitative and volumetric parameters for the different threshold methods at different timing as absolute values (Table 2) as well as variation from PET1 to PET2 and PET3 (Table 3). The scatter plots for the linear regression analyses and the Bland–Altman plots for baseline SUVmean, MTV and TLG values related to the different segmentation techniques used are also displayed (Figure 2, Figure 3 and Figure 4). Among the different methods investigated, the strongest correlation was observed between AM and Vliver (rho > 0.9; *p* < 0.001 for SUVmean, MTV and TLG at all scan timing), along with V2.5 and AM or Vliver (rho 0.98, *p* < 0.001 for TLG at baseline; rho > 0.9; *p* < 0.001 for SUVmean, MTV and TLG at PET2 and PET3).

The standard deviations (SD) according to Bland–Altman plots for baseline SUVmean resulted in favor of the AM and Vliver methods (−1.6 and 1.4), followed by V2.5 and AM or Vliver (Figure 2). The widest range in SD was observed in V41%, with respect to all other methods used.

Similar results were obtained for baseline MTV and TLG (Figure 3 and Figure 4) as well as for all semi-quantitative and volumetric analyses considered at different timing (Supplementary Tables S1 and S2).

To determine the best segmentation method, we applied logistic regression and correlated different results with various Deauville scores obtained at late evaluation (PET3). The results are illustrated as absolute values (Table 4) as well as variations from baseline (Table 5).

Logistic regression demonstrated that MTV and TLG computation according to V2.5 and Vliver significantly correlated to PET3 (*p* = 0.01 and 0.04 for MTV and 0.03 and 0.04 for TLG, respectively), especially when used as absolute values for both DS 2 and DS 3 responses.

SUVmean absolute values were also associated with the responses for Vliver and V41% in the case of DS 2, while as variations, ΔSUVmean correlated to DS 3 for all methods, with the most statistically significant correlation for DS 2 in the case of the V2.5 threshold method (Table 5).

## 4. Discussion

In the last years, several strategies for semi-automatic tumor contouring have been proposed, including fixed (or relative), adaptive or gradient-based (growth of the adaptive region) thresholds. A common unified segmentation method is difficult to develop but necessary in order to improve interinstitutional comparison, find the best reproducibility between semiquantitative and volumetric parameters and ensure optimal patient management within medical centers [38]. However, a consensus on the choice of thresholds has not been reached and the optimal method for tumor volume segmentation is still debated.

Therefore, different methodologies have been studied. In their study, Song et al. [21] enrolled patients with early-stage HL (I-II) and performed their analyses using a threshold of 2.5 as optimal cut-off, demonstrating a correlation between disease prognosis and MTV status. Kanoun et al. [39] investigated the influence of software tools and the total metabolic tumor volume (TMTV) calculation method on prognostic stratification of baseline [^18^F]FDG PET in newly diagnosed HL. They used 2.5, 41%, 125% liver SUVmax and 140% liver SUVmax, proving no significant difference between the respective ROC curves and with optimal cut-offs used being predictive of treatment failure. Martín-Saladich et al. [40] showed an optimal reproducibility in MTV computation for SUV > 2.5 threshold using contouring methodology or software tools. Although the authors found an overestimation of MTV when using a threshold of 2.5, it seemed preferable to the underestimation obtained with the cut-offs of 41% and 50%, respectively. Parvez et al. [41] have reported that the use of a fixed threshold of SUV3 or SUV6 was the best predictor of response to first-line therapy and overall survival.

Eude et al. [42] have compared the reproducibility of MTV measurement as well as the thresholds obtained for each method and their prognostic values in this regard. Eight methods were compared: three absolute thresholds (SUV ≥ 2.5; SUV≥ liver SUVmax; SUV≥ PERCIST SUV), one percentage SUV threshold method (SUV ≥ 41% SUVmax) and four adaptive methods (Daisne, Nestle, Fitting, Black). There was a strong correlation between MTV and patient prognosis regardless of the segmentation method used (*p* = 0.001 for PFS and OS). The largest inter-observer cut-off variability was observed in the 41% SUVmax method, which resulted in more inter-observer disagreements. MTV measurements based on absolute SUV criteria were found to be significantly more reproducible than those based on 41% SUVmax criteria. Recently, Driessen et al. [43] analyzed 105 PET/CT scans from patients with newly diagnosed and relapsed/refractory cHL with six segmentation methods: two fixed thresholds (SUV4.0 and SUV2.5), two relative methods (41% of SUVmax and a contrast-corrected 50% of SUVpeak) and two combination majority vote’ methods (MV2 and MV3). They observed that SUV4.0 tended to underestimate MTV and often missed small lesions, whereas SUV2.5 most frequently included all lesions and generally overestimated MTV. In contrast, few lesions were missed with use of relative or combined thresholds, but these segmentation methods required extensive manual adaptation and overestimated MTV in most cases. There were no significant differences in prognostic performance for all features among the methods.

In our study, we analyzed four segmentation methods: fixed 41% threshold of the SUVmax within the respective lymphoma site (V41%), fixed absolute SUV threshold of 2.5 (V2.5), SUVmax(lesion)/SUVmean liver > 1.5 (Vliver) and adaptive method (AM). With the exception of some outliers, Bland–Altman plots revealed no systematic errors between the different measurement approaches. The calculated limits of agreement were substantial, especially for the V41% compared to the other methods. Consequently, the best correlation for volumetric analysis was documented for AM and Vliver, followed by V2.5. Moreover, the volumetric analysis obtained from V2.5 and Vliver significantly correlated to response to therapy, proving to be preferred thresholds in our pediatric HL cohort.

The results of our study are in line with the cited studies currently in the literature and even when there were no significant differences between the main segmentation methods studied, the absolute SUV method appeared statistically as the most robust. In fact, in many studies [21,39,40,42,43], MTV for SUV > 2.5 threshold has shown an optimal reproducibility and a good correlation with prognosis.

One of the main limitations of our study is the use of images obtained from different scanners and subjected to different algorithm reconstructions. The parameters extracted could lose out from this bias, although harmonization based on EANM Research Ltd. (EARL, Vienna, Austria) accreditation is recommended for experimentation [35]. However, this is a preliminary and parallel study to the prospective study sponsored by the AIEOP Hodgkin Lymphoma Study Group, aimed at investigating the role of volumetric and texture (radiomic) characteristics better fulfilling the need for predictive and prognostic factors in pediatric HL.

## 5. Conclusions

Volumetric analyses with [^18^F]FDG PET/CT are known to help predict the outcome in adult patients with lymphoma. This suggests a similar implication for the pediatric population with HL. To better define the optimal method for tumor volume segmentation, we performed a direct comparison of four computations based on either fixed thresholds (i.e., V41%, V2.5, Vliver) or adaptive methods (AM). Based on our findings, the best correlation for volumetric analysis was documented for AM and Vliver, followed by V2.5. The volumetric analyses obtained from V2.5 and Vliver significantly correlated to response to therapy, proving to be the preferred thresholds for volumetric analyses in our pediatric HL cohort.

## Figures and Tables

**Table 1:** Principal characteristics of the study population.

**Characteristics**	**Number (%)**
**Sex**	
Male	31 (62%)
Female	19 (38%)
**Age (years)**	14.4
Range	7–25
**Stage**	
II	20 (40%)
III	8 (16%)
IV	22 (44%)
**Bulky masses**	30 (60%)
**B symptoms**	25 (50%)
Treatment levels (TLs) *	
TL-1	0 (0%)
TL-2	21 (42%)
TL-3	29 (58%)

* Patients are stratified at baseline into treatment levels according to stage and risk factors, confirmed by central review: TL-1, TL-2 and TL-3 for low, intermediate and advanced HL, respectively.

**Table 2:** Comparison of different absolute threshold values by means of linear regression.

	PET1						PET2						PET3					
Thresholds	SUVmean		MTV		TLG		SUVmean		MTV		TLG		SUVmean		MTV		TLG	
	*rho*	*p*	*rho*	*p*	*rho*	*p*	*rho*	*p*	*rho*	*p*	*rho*	*p*	*rho*	*p*	*rho*	*p*	*rho*	*p*
2.5 vs. 41%	0.83	<0.001	0.36	0.011	0.88	<0.001	0.97	<0.001	0.31	0.037	0.42	0.009	0.88	<0.001	0.90	<0.001	0.96	<0.001
2.5 vs. AM	0.79	<0.001	0.47	0.001	0.98	<0.001	0.93	<0.001	0.91	<0.001	0.96	<0.001	0.99	<0.001	0.98	<0.001	1	<0.001
2.5 vs. liver	0.81	<0.001	0.48	<0.001	0.98	<0.001	0.87	<0.001	0.91	<0.001	0.95	<0.001	0.98	<0.001	0.99	<0.001	1	<0.001
AM vs. liver	0.90	<0.001	0.94	<0.001	0.98	<0.001	0.92	<0.001	0.95	<0.001	0.97	<0.001	0.99	<0.001	0.99	<0.001	1	<0.001
41% vs. AM	0.80	<0.001	0.65	<0.001	0.86	<0.001	0.93	<0.001	0.45	0.001	0.5	0.001	0.88	<0.001	0.95	<0.001	0.97	<0.001
41% vs. liver	0.77	<0.001	0.70	<0.001	0.89	<0.001	0.87	<0.001	0.39	0.006	0.47	0.002	0.91	<0.001	0.92	<0.001	0.96	<0.001

**Table 3:** Comparison of different delta threshold values by means of linear regression.

	Δ PET2						Δ PET3					
Thresholds	ΔSUVmean		ΔMTV		ΔTLG		ΔSUVmean		ΔMTV		ΔTLG	
	*rho*	*p*	*rho*	*p*	*rho*	*p*	*rho*	*p*	*rho*	*p*	*rho*	*p*
2.5 vs. 41%	0.83	<0.001	0.19	0.198	0.37	0.008	0.90	<0.001	0.92	<0.001	0.91	<0.001
2.5 vs. AM	0.88	<0.001	0.93	<0.001	0.97	<0.001	0.96	<0.001	0.94	<0.001	0.98	<0.001
2.5 vs. liver	0.88	<0.001	0.94	<0.001	0.97	<0.001	0.93	<0.001	0.98	<0.001	0.98	<0.001
AM vs. liver	0.94	<0.001	0.95	<0.001	0.97	<0.001	0.94	<0.001	0.93	<0.001	0.97	<0.001
41% vs. AM	0.89	<0.001	0.29	0.040	0.45	0.001	0.90	<0.001	0.82	<0.001	0.91	<0.001
41% vs. liver	0.88	<0.001	0.14	0.345	0.37	0.008	0.89	<0.001	0.96	<0.001	0.97	<0.001

**Table 4:** Logistic regression of different baseline threshold values with respect to post-treatment Deauville score.

		DS 2			DS 3			DS 4			DS 5		
Thresholds		*p*	Odds Ratio	95% CI	*p*	Odds Ratio	95% CI	*p*	Odds Ratio	95% CI	*p*	Odds Ratio	95% CI
2.5	SUVmean	0.37	1.3	0.7–2.3	0.01	1.4	0.9–2	0.01	1.6	1–2.6	0.77	1	0.7–1.7
	MTV	0.01	1	0.9–1	0.07	1	0.9–1	0.02	1	0.9–1	0.9	1	0.9–1
	TLG	0.03	1	0.9–1	0.2	1	0.9–1	0.08	1	0.9–1	0.7	1	0.9–1
41%	SUVmean	0.03	1.1	0.9–1.5	0.1	1.1	0.9–1.4	0.005	1.3	1–1.6	0.3	1.1	0.9–1.4
	MTV	0.14	1	0.9–1	0.8	1	0.9–1	0.7	1	0.9–1	0.6	0.9	0.9–1
	TLG	0.13	1	0.9–1	0.4	1	0.9–1	0.25	1	0.9–1	0.7	0.9	0.9–1
Liver	SUVmean	0.3	1.3	0.7–2.3	0.07	1.4	0.9–2.1	0.02	1.6	1–2.4	0.3	1.2	0.8–1.9
	MTV	0.04	1	0.9–1	0.4	1	0.9–1	0.2	1	0.9–1	0.5	1	0.9–1
	TLG	0.04	1	0.9–1	0.3	1	0.9–1	0.1	1	0.9–1	0.7	1	0.9–1
AM	SUVmean	0.2	1.4	0.8–2.4	0.06	1.4	0.9–2	0.01	1.6	1–2.3	0.1	1.3	0.8–2
	MTV	0.08	1	0.9–1	0.7	1	0.9–1	0.4	1	0.9–1	0.8	1	0.9–1
	TLG	0.05	1	0.9–1	0.3	1	0.9–1	0.15	1	0.9–1	0.9	1	0.9–1

**Table 5:** Logistic regression of different delta threshold values with respect to post-treatment Deauville scores.

		DS 2			DS 3			DS 4			DS 5		
Thresholds		*p*	Odds Ratio	95% CI	*p*	Odds Ratio	95% CI	*p*	Odds Ratio	95% CI	*p*	Odds Ratio	95% CI
2.5	ΔSUVmean	<0.0001	0.4	-	0.0004	0.9	0.94–0.98	0.0005	0.9	0.95–0.99	0.009	0.9	0.92–0.99
	ΔMTV	-	-	-	0.2	0.9	0.9–1	0.09	0.9	0.9–1	0.8	0.9	0.9–1
	ΔTLG	-	-	-	0.1	0.9	0.9–1	0.07	0.9	0.8–1	0.75	0.9	0.9–1
41%	ΔSUVmean	-	-	-	0.0009	0.9	0.92–0.98	0.007	0.9	0.94–0.99	0.008	0.9	0.94–0.99
	ΔMTV	-	-	-	0.5	0.9	0.9–1	0.3	0.9	0.98–1	0.8	1	0.98–1
	ΔTLG	-	-	-	0.9	1	0.9–1	0.9	0.9	0.97–1	0.9	0.9	0.97–1
Liver	ΔSUVmean	-	-	-	0.004	0.9	0.95–0.99	0.01	0.97	0.95–0.99	0.05	0.97	0.95–1
	ΔMTV	-	-	-	0.09	0.9	0.8–1	0.04	0.9	0.8–1	0.3	0.97	0.92–1
	ΔTLG	-	-	-	0.1	0.9	0.8–1	0.06	0.9	0.8	0.4	0.97	0.92–1
AM	ΔSUVmean	-	-	-	0.01	0.9	0.95–0.99	0.03	0.9	0.95–0.99	0.07	0.97	0.95–1
	ΔMTV	-	-	-	0.1	0.9	0.9–1	0.05	0.9	0.9–1	0.6	0.98	0.94–1
	ΔTLG	-	-	-	0.1	0.9	0.9–1	0.07	0.9	0.8–1	0.7	0.98	0.94–1

## Data Availability

Not applicable.

## Supplementary Materials

The following supporting information can be downloaded at: https://www.mdpi.com/article/10.3390/jcm11206223/s1, Table S1: Comparison of different absolute threshold values by means of Bland-Altman method; Table S2: Comparison of different delta threshold values by means of Bland-Altman method; Table S3: List of AIEOP Centers and corresponding investigators involved in the study.

## References

[1] Swerdlow A.J. (2003). Epidemiology of Hodgkin’s disease and non—Hodgkin’s lymphoma. Eur. J. Nucl. Med. Mol. Imaging.

[2] Hudson M.M., Krasin M.J., Kaste S.C. (2004). PET imaging in pediatric Hodgkin’s lymphoma. Pediatr. Radiol..

[3] Kleis M., Daldrup-Link H., Matthay K., Goldsby R., Lu Y., Schuster T., Schreck C., Chu P.W., Hawkins R.A., Franc B.L. (2009). Diagnostic value of PET/CT for the staging and restaging of pediatric tumors. Eur. J. Nucl. Med. Mol. Imaging.

[4] Riad R., Omar W., Kotb M., Hafez M., Sidhom I., Zamzam M., Zaky I., Abdel-Dayem H. (2010). Role of PET/CT in malignant pediatric lymphoma. Eur. J. Nucl. Med. Mol. Imaging.

[5] Franzius C., Juergens K.U., Vormoor J. (2006). PET/CT with diagnostic CT in the evaluation of childhood sarcoma. AJR.

[6] McCarville B. (2006). The role of positron emission tomography in pediatric musculoskeletal oncology. Skeletal. Radiol..

[7] Sharp S.E., Shulkin B., Gelfand M.J., Salisbury S., Kim E.E. (2009). 123I-MIBG scintigraphy and 18F-FDG PET in neuroblastoma. J. Nucl. Med..

[8] Lopci E., Burnelli R., Ambrosini V., Nanni C., Castellucci P., Biassoni L., Rubello M., Fanti S. (2008). (18)F-FDG PET in Pediatric Lymphomas: A Comparison with Conventional Imaging. Cancer Biother. Radiopharm..

[9] Lopci E., Burnelli R., Guerra L., Cistaro A., Piccardo A., Zucchetta P., Derenzini E., Todesco A., Garaventa A., Schumacher F. (2011). Postchemotherapy PET evaluation correlates with patient outcome in paediatric Hodgkin’s disease. Eur. J. Nucl. Med. Mol Imaging.

[10] Lopci E., Mascarin M., Piccardo A., Castello A., Elia C., Guerra L., Borsatti E., Sala A., Todesco A., AIEOP Hodgkin Lymphoma Study Group, Italy (2019). FDG PET in response evaluation of bulky masses in paediatric Hodgkin’s lymphoma (HL) patients enrolled in the Italian AIEOP-LH2004 trial. Eur. J. Nucl. Med. Mol. Imaging.

[11] Cheson B.D., Fisher R.I., Barrington S.F., Cavalli F., Schwartz L.H., Zucca E., Lister T.A. (2014). Recommendations for initial evaluation, staging, and response assessment of Hodgkin and non-Hodgkin lymphoma: The Lugano classification. J. Clin. Oncol..

[12] Barrington S.F., Mikhaeel N.G., Kostakoglu L., Meignan M., Hutchings M., Müeller S.P., Schwartz L.H., Zucca E., Fisher R.I., Trotman J. (2014). Role of imaging in the staging and response assessment of lymphoma: Consensus of the International Conference on malignant lymphomas imaging Working group. J. Clin. Oncol..

[13] Furth C., Amthauer H., Hautzel H., Steffen I.G., Ruf J., Schiefer J., Schönberger S., Henze G., Grandt R., Hundsdoerfer P. (2011). Evaluation of interim PET response criteria in paediatric Hodgkin’s lymphoma—Results for dedicated assessment criteria in a blinded dual-centre read. Ann. Oncol..

[14] Kluge R., Chavdarova L., Hoffmann M., Kobe C., Malkowski B., Montravers F., Kurch L., Georgi T., Dietlein M., Wallace W.H. (2016). Inter-Reader Reliability of Early FDG-PET/CT Response Assessment Using the Deauville Scale after 2 Cycles of Intensive Chemotherapy (OEPA) in Hodgkin’s Lymphoma. PLoS ONE.

[15] Barrington S.F., Kluge R. (2017). FDG PET for therapy monitoring in Hodgkin and non-Hodgkin lymphomas. Eur. J. Nucl. Med. Mol. Imaging.

[16] Hasenclever D., Kurch L., Mauz-Körholz C., Georgi T., Wallace H., Landman-Parker J., Moryl-Bujakowska A., Cepelová M., Karlén J., Fernández-Teijeiro A.Á. (2014). qPET—A quantitative extension of the Deauville scale to assess response in interim FDG-PET scans in lymphoma. Eur. J. Nucl. Med. Mol. Imaging.

[17] Mauz-Körholz C., Hasenclever D., Dörffel W., Ruschke K., Pelz T., Voigt A., Stiefel M., Winkler M., Vilser C., Dieckmann K. (2010). Procarbazine-free OEPA-COPDAC chemotherapy in boys and standard OPPA-COPP in girls have comparable effectiveness in pediatric Hodgkin’s lymphoma: The GPOH-HD-2002 study. J. Clin. Oncol..

[18] Rogasch J.M.M., Hundsdoerfer P., Hofheinz F., Wedel F., Schatka I., Amthauer H., Furth C. (2018). Pretherapeutic FDG-PET total metabolic tumor volume predicts response to induction therapy in pediatric Hodgkin’s lymphoma. BMC Cancer.

[19] Mauz-Körholz C., Landman-Parker J., Balwierz W., Ammann R.A., Anderson R.A., Attarbaschi A., Bartelt J.M., Beishuizen A., Boudjemaa S., Cepelová M. (2022). Response-adapted omission of radiotherapy and comparison of consolidation chemotherapy in children and adolescents with intermediate-stage and advanced-stage classical Hodgkin lymphoma (EuroNet-PHL-C1): A titration study with an open-label, embedded, multinational, non-inferiority, randomised controlled trial. Lancet Oncol..

[20] Akhtari M., Milgrom S.A., Pinnix C.C., Reddy J.P., Dong W., Smith G.L., Mawlawi O., Yehia Z.A., Gunther J., Osborne E.M. (2018). Re-classifying patients with early-stage Hodgkin lymphoma based on functional radiographic markers at presentation. Blood.

[21] Song M.-K., Chung J.-S., Lee J.-J., Jeong S.Y., Lee S.-M., Hong J.-S., Chong A., Moon J.-H., Kim J.-H., Lee S.-M. (2013). Metabolic tumor volume by positron emission tomography/computed tomography as a clinical parameter to determine therapeutic modality for early stage Hodgkin’s lymphoma. Cancer Sci..

[22] Kim J., Hong J., Kim S.G., Hwang K.H., Kim M., Ahn H.K., Sym S.J., Park J., Cho E.K., Shin D.B. (2014). Prognostic value of metabolic tumor volume estimated by (18) F-FDG positron emission tomography/computed tomography in patients with diffuse large B-cell lymphoma of stage II or III disease. Nucl. Med. Mol. Imaging..

[23] Kim T.M., Paeng J.C., Chun I.K., Keam B., Jeon Y.K., Lee S.-H., Kim D.-W., Lee D.S., Kim C.W., Chung J.-K. (2013). Total lesion glycolysis in positron emission tomography is a better predictor of outcome than the International Prognostic Index for patients with diffuse large B cell lymphoma. Cancer.

[24] Cottereau A.S., Versari A., Luminari S., Dupuis J., Chartier L., Casasnovas R.-O., Berriolo-Riedinger A., Menga M., Haioun C., Tilly H. (2018). Prognostic model for high-tumor-burden follicular lymphoma integrating baseline and end-induction PET: A LYSA/FIL study. Blood.

[25] Cottereau A.S., Versari A., Loft A., Casasnovas O., Bellei M., Ricci R., Bardet S., Castagnoli A., Brice P., Raemaekers J. (2018). Prognostic value of baseline metabolic tumor volume in early-stage Hodgkin lymphoma in the standard arm of the H10 trial. Blood.

[26] Albano D., Bosio G., Bianchetti N., Pagani C., Alessandro R., Tucci A., Giubbini R., Bertagna F. (2019). Prognostic role of baseline 18FFDG PET/CTmetabolic parameters in mantle cell lymphoma. Ann. Nucl. Med..

[27] Albano D., Bertoli M., Battistotti M., Rodella C., Statuto M., Giubbini R., Bertagna F. (2018). Prognostic role of pretreatment 18F-FDG PET/CT in primary brain lymphoma. Ann. Nucl. Med..

[28] Albano D., Mazzoletti A., Spallino M., Muzi C., Zilioli V.R., Pagani C., Tucci A., Rossetti C., Giubbini R., Bertagna F. (2020). Prognostic role of baseline 18F-FDG PET/CT metabolic parameters in elderly HL: A two-center experience in 123 patients. Ann. Hematol..

[29] Albano D., Bosio G., Pagani C., Re A., Tucci A., Giubbini R., Bertagna F. (2019). Prognostic role of baseline 18F-FDG PET/CT metabolic parameters in Burkitt lymphoma. Eur. J. Nucl. Med. Mol. Imaging.

[30] Sasanelli M., Meignan M., Haioun C., Berriolo-Riedinger A., Casasnovas R.-O., Biggi A., Gallamini A., Siegel B.A., Cashen A.F., Véra P. (2014). Pretherapy metabolic tumour volume is an independent predictor of outcome in patients with diffuse large B-cell lymphoma. Eur. J. Nucl. Med. Mol. Imaging.

[31] Esfahani S.A., Heidari P., Halpern E.F., Hochberg E.P., Palmer E.L., Mahmood U. (2013). Baseline total lesion glycolysis measured with (18)F-FDG PET/CT as a predictor of progression-free survival in diffuse large B-cell lymphoma: A pilot study. Am. J. Nucl. Med. Mol. Imaging.

[32] Mikhaeel N.G., Smith D., Dunn J.T., Phillips M., Møller H., Fields P.A., Wrench D., Barrington S.F. (2016). Combination of baseline metabolic tumour volume and early response on PET/CT improves progression-free survival prediction in DLBCL. Eur. J. Nucl. Med. Mol. Imaging.

[33] Meignan M., Cottereau A.S., Versari A., Chartier L., Dupuis J., Boussetta S., Grassi I., Casasnovas R.-O., Haioun C., Tilly H. (2016). Baseline Metabolic Tumor Volume Predicts Outcome in High-Tumor-Burden Follicular Lymphoma: A Pooled Analysis of Three Multicenter Studies. J. Clin. Oncol..

[34] Second International Inter-Group Study for Classical Hodgkin Lymphoma in Children and Adolescents. https://clinicaltrials.gov/ct2/show/NCT02684708.

[35] Boellaard R., Delgado-Bolton R., Oyen W.J.G., Giammarile F., Tatsch K., Eschner W., Verzijlbergen F.J., Barrington S.F., Pike L.C., Weber W.A. (2015). FDG PET/CT: EANM procedure guidelines for tumour imaging—Version 2.0. Eur. J. Nucl. Med. Mol. Imaging.

[36] Lopci E., Burnelli R., Elia C., Piccardo A., Castello A., Borsatti E., Zucchetta P., Cistaro A., Mascarin M. (2021). Additional value of volumetric and texture analysis on FDG PET assessment in paediatric Hodgkin lymphoma: An Italian multicentric study protocol. BMJ Open.

[37] Meignan M., Gallamini A., Meignan M., Haioun C. (2009). Report on the First International Workshop on Interim-PET-Scan in Lymphoma. Leuk. Lymphoma..

[38] Barrington S.F., Meignan M. (2019). Time to prepare for risk adaptation in lymphoma by standardizing measurement of metabolic tumor burden. J. Nucl. Med..

[39] Kanoun S., Tal I., Berriolo-Riedinger A., Rossi C., Riedinger J.-M., Vrigneaud J.-M., Legrand L., Humbert O., Casasnovas O., Brunotte F. (2015). Influence of software tool and methodological aspects of total metabolic tumor volume calculation on baseline [18F] FDG PET to predict survival in Hodgkin lymphoma. Clin. Trial.

[40] Martín-Saladich Q., Reynés-Llompart G., Sabaté-Llobera A., Palomar-Muñoz A., Domenech E.D., Cortés-Romera M. (2020). Comparison of different automatic methods for the delineation of the total metabolic tumor volume in I-II stage Hodgkin Lymphoma. Sci. Rep..

[41] Parvez A., Tau N., Hussey D., Maganti M., Metser U. (2018). 18F-FDG PET/CT metabolic tumor parameters and radiomics features in aggressive non-Hodgkin’s lymphoma as predictors of treatment outcome and survival. Ann. Nucl. Med..

[42] Eude F., Toledano M.N., Vera P., Tilly H., Mihailescu S.-D., Becker S. (2021). Reproducibility of Baseline Tumour Metabolic Volume Measurements in Diffuse Large B-Cell Lymphoma: Is There a Superior Method?. Metabolites.

[43] Driessen J., Zwezerijnen G.J., Schöder H., Drees E.E., Kersten M.J., Moskowitz A.J., Moskowitz C.H., Eertink J.J., de Vet H.C., Hoekstra O.S. (2022). The impact of semi-automatic segmentation methods on metabolic tumor volume, intensity and dissemination radiomics in 18F-FDG PET scans of patients with classical Hodgkin lymphoma. J. Nucl. Med..

