# Supplementary material for: Prospective Evaluation of Different Methods for Volumetric Analysis on [18F]FDG PET/CT in Pediatric Hodgkin Lymphoma
Source: J Clin Med. 2022 Oct 21;11(20):6223. doi: 10.3390/jcm11206223 (PMC9605658; doi:10.3390/jcm11206223)
Supplement: Supplementary file 1 [file jcm-11-06223-s001.zip › jcm-1916871-supplementary.pdf]

Table S1. Comparison of different absolute threshold values by means of Bland-Altman method.

	PET1			PET2			PET3		
**Thresholds**	**SUVmean**	**MTV**	**TLG**	**SUVmean**	**MTV**	**TLG**	**SUVmean**	**MTV**	**TLG**
2.5 vs 41%	-3.3	447.6	1440.1	-0.1	-1.5	13.8	0	-7.3	-6
2.5 vs AM	0.3	-18.5	-185.5	0.25	-7.4	-16.6	0.17	-5.2	-13
2.5 vs liver	0.2	28.1	-80.6	-0.13	2.5	12.9	0	1.8	2.7
AM vs liver	-0.1	46.6	104.8	-0.36	9.3	27.3	-0.17	7	15.8
41% vs AM	3.6	-466.1	-2406.3	0.3	-5.1	-20.3	0.2	2.1	-6.9
41% vs liver	3.5	-419.5	-1520.7	0	4.4	7	0	9.1	8.6

Table S2. Comparison of different delta threshold values by means of Bland-Altman method.

	**Δ PET2**			**Δ PET3**		
**Thresholds**	**ΔSUVmean**	**ΔMTV**	**ΔTLG**	**ΔSUVmean**	**ΔMTV**	**ΔTLG**
2.5 vs 41%	-14.7	28.7	6.3	-10.3	44.6	15.3
2.5 vs AM	**2.6**	**0.8**	**0.5**	-1	1	0.4
2.5 vs liver	6.4	-1.3	-0.9	**0.4**	1	0.5
AM vs liver	3.8	-2	-1.3	1.4	**0**	**0.1**
41% vs AM	17.3	-28	-5.8	9.2	-43.6	-15
41% vs liver	21.1	-30	-7.1	10.7	-43.7	-14.9

Table S3. List of AIEOP Centers and corresponding investigators involved in the study.

CENTER/CITY	DEPARTMENT	LOCAL INVESTIGATOR/ P.I.	NUCLEAR MEDICINE PHYSICIAN
**AVIANO-PORDENONE (CENTRO COORDINATORE)**	IRCCS C.R.O. Aviano - Centro Integrato di EmatoOncologia Pediatrica e dell'adolescenza	Dr. Maurizio Mascarin	**DR. EUGENIO BORSATTI**
**TORINO**	S.C. Oncoematologia Pediatrica e Centro Trapianti - A.O.U. Città della salute e della Scienza di Torino - Presidio Infantile Regina Margherita	Dr. Franca Fagioli	**PROF DESIRÈE DEANDREIS**
**GENOVA**	Dipartimento funzionale di Emato-oncologia Pediatrica IRCCS "G.Gaslini"	Dr. Alberto Garaventa	**DR.SSA ANGELINA CISTARO**
**MONZA**	Clinica Pdiatrica universitaria-Fondazione MBBM A.O. San Gerardo di Monza	Dr. Carmelo Rizzari	**DR. LUCA GUERRA**
**PAVIA**	S.C. Oncoematologia Pediatrica Fondazione Irccs, Policlinico San Matteo	Dr. Marco Zecca	**DR GIORGIO CAVENAGHI**
**BERGAMO**	U.S.S. Oncoematologia Pediatrica - Ospedale Papa Giovanni XXIII	Dr. Massimo Provenzi	**DR LUIGI FRANCO CAZZANIGA**
**BRESCIA**	Oncoematologia pediatrica e TMO - Spedali Civili di Brescia - Ospedale dei Bambini	Dr. Fulvio Porta	**DR. MATTIA BERTOLI**
**PADOVA**	Oncoematologia Pediatrica - A.O.U. di Padova	Dr.ssa Marta Pillon	**DR. PIETRO ZUCCHETTA**
**VERONA**	U.O.C Oncoematologia Pediatrica - Policlinico "G.B. Rossi" - AOUI Verona	Dr. Simone Cesaro	**DR. MICHELE ZUFFANTE**
**TRIESTE**	S.C. Onco-ematologia Pediatrica, S.S. Trapianto di Midollo - Dipartimento pediatrico IRCCS Materno Infantile "Burlo Garofolo "	Dr. Federico Verzegnassi	**DR. EUGENIO BORSATTI**
**PARMA**	U.O.C di Pediatria e Oncoematologia - AOU di Parma	Dr.ssa Patrizia Bertolini	**DR.SSA LIVIA RUFFINI**
**MODENA**	Pediatria ad indirizzo oncoematologico - Azienda Policlinico di Modena	Dr.ssa Cellini Monica	**DR.SSA ANTONELLA FRANCESCHETTO**

**BOLOGNA**	Oncologia ed Ematologia Pediatrica "Lalla Seragnoli" Clinica Pediatrica Policlinico Sant'Orsola Malpighi	Dr.ssa Elena Facchini	**PROF. STEFANO FANTI**
**FERRARA**	SSD Oncoematologia Pediatrica - AOU "Sant'Anna di Ferrara	Dr.ssa Roberta Burnelli	**DR.SSA ILARIA RAMBALDI**
**RIMINI**	U.O. di Pediatria, SS Oncoematologia PediatricaOspedale Infermi di Rimini	Dr.ssa Roberta Pericoli	**DR.SSA FEDERICA MATTEUCCI**
**FIRENZE**	Dipartimento Oncoematologia SODC Tumori Pediatrici e Trapianto di cellule staminali, A.O.U "Anna Meyer"	Dr. Tommaso Casini	**DR. KATIA OLIANTI**
**SIENA**	Clinica Pediatrica - Policlinico "Le Scotte" - Siena	Dr.ssa Daniela Galimberti	**DR. CATIA OLIANTI**
**PISA**	U.O. Oncoematologia Pediatrica- AOU Pisana - Ospedale S. Chiara	Dott.ssa Gabriella Casazza	**DR. DUCCIO VOLTERRANI**
**PERUGIA**	S.C. di Oncoematologia Pediatrica con Trapianto di CSE, AOU 'S.M. della Misericordia'.di Perugia	Dr. Maurizio Caniglia	**DR. SALVATORE MESSINA**
**ANCONA**	SOS Oncoematologia Pediatrica - Azienda Ospedali Riuniti Presidio "G. Salesi "	Dr.ssa Irene D'Alba	**DR.SSA GIOVANNA MANTELLO**
**PESCARA**	Dipartimento di Ematologia, medicina trasfusionale e biotecnologie- Presidio Ospedale Civile dello Spirito Santo Pescara Dr.ssa Antonella Sau	Dr.ssa Antonella Sau	**DR. CARLO VILLANO**
**ROMA**	UOS Ematologia Pediatrica - Policlinico Umberto I - Università "La Sapienza" di Roma	Dr.ssa Anna Maria Testi	**DR.SSA LUCIA LECCISOTTI**
**ROMA**	Dipartimento di Ematologia Oncologia e medicina trasfusionale IRCCS Ospedale Pediatrico "Bambino Gesù "	Prof. Franco Locatelli	**DR.SSA LUCIA LECCISOTTI**
**NAPOLI UNIVERSITÀ**	A.O.U. Vanvitelli U.O.S.D. ematologia e oncologia pediatrica	Dr.ssa Francesca Rossi	**DR. PAOLO GABALLO**

**NAPOLI PAUSILIPON**	Dipartimento di Oncoematologia A.O.R.N. Santobono Pausilipon	Dr. Salvatore Buffardi	**DR. CIRO MAINOLFI**
**SAN GIOVANNI ROTONDO**	UOC Oncoematologia Pediatrica IRCCS Ospedale "Casa Sollievo Della Sofferenza" S.G. Rotondo	Dr.ssa Raffaella De Santis	**DR. ANTONIO CANISTRO**
**TARANTO**	Ospedale SS. Annunziata UOC Pediatria e Oncoematologia Pediatrica	Dr. Valerio Cecinati	**DR. ANTONIO NOTARISTEFANO**
**LECCE**	UOC Oncoematologia pediatrica - PO "Vito Fazzi"	Dr.ssa Assunta Tornesello	**DR.SSA ELISA CAVALERA**
**BARI**	Dipartimento di Pediatria - AOU Policlinico di Bari	Dr. Nicola Santoro	**DR.SSA CRISTINA FERRARI**
**CATANZARO**	UOC Ematologia ed Oncologia Pediatrica - AO "Pugliese-Ciaccio "	Dr.ssa Maria Concetta Galati	**DR. PAOLO PUNTIERI**
**COSENZA**	UOC Pediatria - SO "Annunziata"	Dr. Domenico Sperlì	**DR. ANTONIO BAGNATO**
**PALERMO**	U.O.C. Oncoematologia Pediatrica - ARNAS Civico di Cristina e Benfratelli	Dr. Piero Farruggia	**DR. MICO OLIVIERI**
**CATANIA**	UOC Ematologia ed Oncologia Pediatrica con TMO - AOU Policlinico "Vittorio Emanuele"	Dr. Salvatore D'Amico	**DR. MASSIMO IPPOLITO**
**SASSARI**	Clinica Pediatrica - AOU Sassari	Dr. Roberto Antonucci	**DR.SSA DANIELA SANNA**
**CAGLIARI**	**SC ONCOEMATOLOGIA PEDIATRICA E PATOLOGIA DELLA COAGULAZIONE - OSPEDALE PEDIATRICO MICROCITEMICO "ANTONIO CAO" - AO BROTZU**	**DR.SSA ROSAMARIA MURA**	**DR. LUCA MELIS**
